# 426. The diagnosis and treatment of COVID Associated Pulmonary Aspergillosis in patients requiring Veno-Venous Extracorporeal Membrane Oxygenation (VV-ECMO) in the Republic of Ireland during the COVID-19 pandemic: A Retrospective Review

**DOI:** 10.1093/ofid/ofad500.496

**Published:** 2023-11-27

**Authors:** Rhea O’Regan, Gillian Crowe, Maureen Lynch, Eavan Muldoon, Jennifer Hastings

**Affiliations:** Mater Misericordiae University Hosptial, Dublin, Ireland, Dublin, Dublin, Ireland; Mater Misericordiae University Hospital, Dublin, Dublin, Ireland; Mater Misericordiae University Hospital, Dublin, Dublin, Ireland; Mater Misericordiae University Hospital, Dublin, Dublin, Ireland; Mater Misericordiae University Hospital, Dublin, Dublin, Ireland

## Abstract

**Background:**

During the advent of the COVID-19 pandemic, multiple cases were reported of the association between SARS-CoV-2 and invasive pulmonary aspergillosis. The diagnosis of Covid associated pulmonary aspergillosis (CAPA) remains difficult, especially in critically unwell patients. We describe those treated with VV ECMO and diagnosed with CAPA in the national centre for ECMO in Ireland during the COVID-19 pandemic, as well as their treatment course and outcomes.

**Methods:**

We retrospectively collected data on all patients who received ECMO during the COVID-19 pandemic in Ireland’s national ECMO centre between May 2020 and January 2022. Data was collected from electronic health care records and cross referenced with positive microbiological samples. The 2020 ECMM/ ISCHAM consensus criteria was used to classify CAPA diagnoses.

**Results:**

37 patients with diagnosed SARS-CoV-2 infection were treated using ECMO. The average age was 46 years (SD 9.3). 15 (41%) of those were female. The mean number of days spent in an intensive care unit (ICU) was 50 (IQR 45-67). 14 (38%) had diagnosed Hypertension, 10 (27%) had Diabetes Mellitus and 1 (2.7%) had known Chronic Kidney Disease requiring dialysis. CAPA was diagnosed in 7 (19%) cases. Using the criteria, no cases were proven as histology samples were not obtained. 1 (14%) case fitted the criteria for possible diagnosis while 6 (86%) cases fitted criteria for probable diagnosis based on biochemical, microbiological and radiological findings. Of these, all received steroids prior to diagnosis, while 3 (43%) also received tociluzumab. 6 (86%) patients received dual antifungal agents and 1 (14%) patient received voriconazole alone. In terms of outcome, 15 (41%) were discharged to another intensive care unit, 1 (3%) discharged to another acute hospital and 2 (5%) to a rehabilitation unit. 16 (43%) patients died while 3 (8%) were discharged home. Of the 7 patients diagnosed with CAPA, 5 (71%) patients died in ICU while 2 (29%) were transferred to other units with final outcome unknown.

**Conclusion:**

Although 2020 guidelines available from ECMM/ISCHAM are used to define strict diagnostic criteria of CAPA, our study demonstrates the need for further research into how other factors may contribute to this diagnosis, including pre-determined risk factors or the use of ECMO.

**Disclosures:**

**All Authors**: No reported disclosures

